# Ahmed Glaucoma Valve Implant: Experience in East Africa

**DOI:** 10.4103/0974-9233.56230

**Published:** 2009

**Authors:** D.O. Kiage, D. Gradin, S. Gichuhi, K.F. Damji

**Affiliations:** From the Department of Surgery, Aga Khan University Hospital, Nairobi, Kenya; 1 From the Department of Eye Unit, PCEA Kikuyu Hospital, Kenya; 2 From the Deparmtnet of Ophthalmology, University of Nairobi, Nairobi, Kenya; 3 From the University of Alberta, Edmonton, Canada

**Keywords:** Aqueous Rainage Devices, Glaucoma Surgery, Intraocular Pressure

## Abstract

**Purpose:**

To describe short term outcomes of Ahmed Glaucoma Valve [AGV] implantation in East African patients.

**Materials and Methods:**

In this multi-center retrospective case series we reviewed eyes of Black African patients with refractory glaucoma, treated consecutively with Ahmed Glaucoma Valve implantation, in two centers in Kenya between January 2006 and October 2007.

**Results:**

About 25 cases including 18 [72%] pediatric eyes and seven [28%] adult eyes were identified. Results have been presented with a median follow-up of two months with inter-quartile range [IQR] of one to 12 months. intraocular pressure [IOP] was reduced from a mean of 36.4 mmHg preoperatively to 16.7 mmHg and glaucoma medications were lowered from a mean of 1.32 before surgery to 0.2 after surgery. The success rate during short term follow-up was 79%. The mean visual acuity dropped slightly from 6/18 pre-operatively to 6/24. There was only one major complication of an extruded, infected valve in a child.

**Conclusions:**

The Ahmed Valve Implant is safe and effective in lowering IOP for the short term in pediatric and adult East African patients with refractory glaucoma. Further studies with more patients and longer term follow-up are needed in this population.

## INTRODUCTION

Glaucoma is a leading cause of blindness worldwide, with a higher prevalence in the Black race, often with greater challenges in diagnosis and management.^1^–^5^ Glaucoma is diagnosed at a more advanced stage, progresses more rapidly and is less responsive to medical treatment in Blacks compared to whites.^6^,^7^ A study in East Africa showed that in a rural village as low as two per cent of the patients with glaucoma had been identified.^8^ Although filtering surgery is generally recommended as the first line treatment for Primary Open Angle Glaucoma [POAG] in Sub-Saharan Africa, the failure rate of surgery is higher in Blacks vs. Whites.^2^ Further, African American patients with glaucoma are more likely to require surgical intervention than white patients [2.95 vs. 1.38 glaucoma surgical procedures per 1000 persons per year].^9^ Possible explanations for these differences include increased proliferation and more vigorous wound healing, lower central corneal thickness and socioeconomic factors.^1^,^4^,^5^,^10^

Glaucoma management is currently under evaluation to determine appropriate recommendations for Sub-Saharan African patients. Glaucoma Drainage Devices [GDDs] are a useful alternative in cases in which medical treatment and filtering surgery have failed, or are likely to fail. Some studies have also recommended the devices for primary procedures in all types of glaucoma, depending on the level of IOP lowering desired, availability of medicines and the comfort level of the surgeon performing the procedure.^11^,^12^ The current study evaluates the initial indications and outcomes of AGV implants performed in the Black population at two institutions in East Africa. The data gleaned from this early experience will form the basis for subsequent randomized controlled trials comparing glaucoma tube surgery and traditional filtering procedures in this population.

## MATERIALS AND METHODS

A retrospective case series design was used. Records of all AGV implants done by three surgeons (DK, DG and KD) in two hospitals (Aga Khan University Hospital and PCEA Kikuyu Hospital) in Kenya between January 2006 and October 2007 were reviewed. Pre-operative information included gender, age, lens status, glaucoma type and status, history of previous eye surgery, number of glaucoma medicines, IOP measured by Goldman applanation tonometry, visual acuity, and optic nerve as well as visual field information if available. The details of surgery, including implant location, eye operated, anesthetic technique, intraoperative complications and model of implant used, were recorded. Postoperative data regarding IOP, number of glaucoma medications, visual acuity and complications were obtained on day 1, day 7, weeks 2 and 4 and thereafter as they were reviewed.

Success was defined as IOP of between six and 21 mmHg or 20% IOP reduction with or without additional medication, without further glaucoma surgery or laser treatment, without devastating complications [e.g. endophthalmitis, suprachoroidal hemorrhage, prolonged hypotony with maculopathy and/or choroidal effusions] and without loss of light perception.

The implant used was AGV [New World Medical Inc., Rancho Cucamonga, California]. Implantation was performed as per standard procedure.^11^ In brief, a fornix-based conjunctival flap was created between the superior and lateral rectus muscles. Adequate cautery to episcleral vessels was done. The tube of the AGV was irrigated by a 26 gauge canula with Balanced Salt Solution or Ringers Lactate to prime the valve. The plate was inserted under the conjunctiva/tenons flap and secured to the sclera about 8mm posterior to the limbus with nine-zero nylon sutures. The tube was trimmed with the bevel facing up, to extend approximately two mm beyond the surgical limbus into anterior chamber. A 23 gauge needle was used to enter the anterior chamber at the surgical limbus parallel to the iris plane. The tube was inserted into the anterior chamber through a needle track using the special inserter or a non-toothed forceps. The tube was then covered with human donor sclera or pericardium which was secured with nine-zero nylon sutures. The human donor sclera was prepared as follows: The sclera was initially stored in a container with absolute alcohol. A 5 × 5 mm piece was cut from the sclera taking care to obtain a piece that was uniformly thick. The piece was soaked in a dish containing 100 cc of Balanced Salt Solution or Ringers Lactate for 15 minutes. The piece was then changed to another similar dish containing 100 cc of Balanced Salt Solution or Ringers Lactate for 15 minutes more. Then the piece was transferred to a dish with 100 cc of similar solution with 80 mg gentamicin for another 15 minutes. The piece was then rinsed and trimmed further to the right size to cover the tube and stitched as indicated above.

The conjunctiva was then closed snugly with 10-0 nylon and sub- conjunctival injection of gentamicin and dexamethasone were administered. A steroid-antibiotic ointment was applied and the eye was patched. On the first postoperative day patients were reviewed and put on topical steroids and antibiotic treatment. The follow-ups were scheduled after one week, one month, and then once every two months for one year and then every four months thereafter.

### Statistical analysis

Descriptive statistics using frequencies and proportions were summarized. Means with standard deviations and/or the 95% confidence intervals of the mean were reported where the data was normally distributed and medians where the distribution was not. The level of significance used was 95%.

## RESULTS

The characteristics of the patients in the study are summarized in Table 1. There were 25 eyes of 20 patients operated. The median follow up period was two months with an interquartile range [IQR] of one to 12 months. There were 18 [72%] children and 7 [28%] adults and their mean ages in years were 3.9 [s.d. 3.4] and 61.0 [s.d. 9.6] respectively. The sex distribution was 21 [84%] male and four [16%] female. There were five previous trabeculectomies, five trabeculotomies, one goniotomy and one combined trabeculectomy and cataract. Other previous eye surgeries included cataract extraction [ECCE/phacoemulsification] with IOL - five, lensectomies – five, lensectomy with IOL, lens wash out and pan-retinal photocoagulation - one each. Among the 25 eyes operated, 11 were diagnosed with congenital glaucoma, four pseudo phakic/aphakic glaucoma, five anterior segment dysgenesis, three POAG and one ocular trauma and one neovascular glaucoma. Twenty operations were done under general anesthesia and five under peri-bulbar anesthesia.

**Table 1:** Patient characteristics

Number of patients	20
Eyes	25
Follow up	
Median	2 months
Interquartile range [IQR]	1-12 months
Age [years]	
Children	Mean – 3.9 [sd 3.4] N – 18
Adults	[72]
	Mean – 61.0 [sd 9.6] N – 7
	[28]
Gender n[%]	M – 21 [84] F – 4 [16]
Previous glaucoma surgeries	
Trabeculectomy	5
Trabeculotomy	5
Goniotomy	1
Combined trab. + CE	1
Other previous eye surgeries	
ECCE/Phaco + IOL	5
Lensectomy	5
Lensectomy + IOL	1
Lens wash out	1
Pan retino photocoagulation	1
Type of glaucoma – n [%]	
Congenital glaucoma	11 (44%)
Pseudophakic/aphakic glaucoma	4 (16%)
Anterior segment dysgenesis	5 (20%)
POAG	3 [12%]
Trauma	1 (4%)
Neovascular glaucoma	1 (4%)
Anesthesia	GA – 20 [80]; LA – 5 [20]

The mean preoperative IOP was 36.4 mmHg, and the mean postoperative IOP at the latest review date was 16.7 mmHg, giving a mean percentage IOP-lowering of 53.2% [Table 2]. A slight spike of IOP was noted on day 30 followed by a subsequent reduction. There was a reduction in the number of antiglaucoma medicines used from 1.32 to 0.2 [Table 3]. The mean visual acuity was 6/18 preoperatively and it dropped to 6/60 on day one after surgery then dropped further to counting fingers on day seven before improving to 6/24 by one month. Success was achieved in 19 [79%] patients and failure in five patients. One patient did not have any IOP taken postoperatively and was not included in the percentage of success calculation. Postoperative complications included hyphaema in three eyes, cosmetically large blebs in three cases, retracted tube in one case, dellen with exposed scleral patch in one patient, post-operative hypotony in one patient, anterior chamber tube blockage with vitreous in one patient and blebitis with extruded implant which led to implant removal in one patient. The other causes for failure included high postoperative IOPs in four eyes of three children who were aged one month, six months and 11 months at the time of surgery.

**Table 2:** Clinical outcomes

	n/%
Anti-glaucoma Medications [n]	
Preoperative	1.32
Postoperative	0.2
Success	19
Failure	5
Percentage	79
Complications	
Hyphaema	3
Cosmetically large blebs	3
AC tube block with vitreous	1
Retracted tube	1
Dellen with exposed scleral patch	1
Blebitis and implant extrusion	1
Hypotony	1

**Table 3:** Success rate comparison with other studies in adults and pediatrics

Study	Success rate %
Huang *et al.*^18^	87 - 1 yr
	75 - 2 yr
Wilson *et al.*^15^	94.4 - D1
	90.7 - D7 to wk 15
	88.1 - 13 mo
Topouzis *et al.*^13^	87 - 1 yr
	82 - 2 yr
	76 - 3-4 yrs
Chen *et al.*^19^ [Pediatric patients]	87.1 - 1 yr, 63.2 - 2 yr
	51.7 - 3 yr
	41.8 - 4 yr
This study	79 - 2 mo

## DISCUSSION

Glaucoma Drainage Devices consist of a silicone tube that extends through the anterior chamber or vitreous cavity to a plate, disc or encircling element beneath the conjunctiva and Tenon's capsule.^13^ The AGV incorporates a valve mechanism which decreases early postoperative hypotony by providing resistance to flow and therefore regulating the pressure within a desired range. Studies have shown that it lowers IOP by between 49-64% on a mean follow-up of 31 months.^11^,^12^,^14^

Although AGV implants have been in use since 1993, and in spite the well-demonstrated indications and high success rates,^12^,^14^–^19^ we were not able to locate published reports on its experience in East Africa. The patients in our study were all Black Africans. Our study included complicated glaucoma cases in which filtering surgeries and/or anterior chamber angle procedures had failed or had very low chances of success. Out of the 25 eyes operated 18 [72%] were in children and seven [28%] in adults; there were more males than females in our series (21 [84%] vs. four [16%]). The glaucoma medications were reduced from a mean of 1.32 to 0.2. The low usage of medicine is characteristic in this region due to their high cost and unavailability. Usually the average medicines usage before surgical will be two to three in a majority of other studies. The other explanation for this low use of medicine was that most of our cases were children for whom the treatment of glaucoma is usually surgical.

Our mean percentage IOP-lowering of 53.2% achieved with the implant compares well with reports in the literature. Lima *et al.* reported a percentage lowering of 64% after a 24 months follow- up in 68 patients.^11^ Tupouzis *et al*. reported 59% IOP- lowering.^12^ In Wilson *et al.* there was a reduction of 35.9% after a 12 month follow-up and 49.4% after 48 month follow-up.^14^,^15^

Our success rate during the study period was 79%. Studies with a similar definition of success have reported the following success rates: Huang *et al.* reported 87% success at one year and 75% success at two years in 159 eyes of 144 patients, including 22 Black, 89 White, 32 Hispanic, and 8 Asian patients.^18^ Wilson *et al.* reported a success of 88% after a follow-up of 13 months. Topouzis *et al.* arrived at rates of 87% at one year, 82% at two years, and 76% at three and four years in 60 patients who consisted of 37 white, 12 Hispanic, eight Black and three Asian.

Studies for AGV in pediatric glaucoma have demonstrated good success rates. Chen *et al.* studied Ahmed valve surgery for refractory pediatric glaucoma with success defined as a reduction of the IOP to 22 mm Hg or lower with or without medications at the last two follow-up visits, no additional glaucoma surgery, and no visually significant complications and found the cumulative probabilities of success were 85.1%, 63.2%, 51.7%, and 41.8% at one, two, three and four years, respectively.^19^ The conclusion from this study by Chen *et al*. was that AGV was useful in the management of refractory pediatric glaucoma. Although the success rates were similar to those observed in adults, the rates of certain post-operative complications were different. Yair Mohad *et al*. used a similar success definition in a retrospective series of 60 eyes and found probability of success of 93% (95% confidence interval [CI], 86%-100%), 86% (95% CI, 77%-96%),71% (95% CI, 59%-87%), and 45% (95% CI, 28%-80%) after 12, 24, 36, and 48 months of follow-up.^20^ In a series of 27 eyes with refractory pediatric glaucoma, Englert *et al.* demonstrated an overall success rate of 85.2% at last follow-up. Cumulative probability of success by Kaplan-Meier analysis was 90.6% at 12 months and 58.3% at 24 months.^21^ According to another study, diagnosis, number of previous glaucoma procedures, and surgeon experience seem to be related to the survival of the AGV implant in pediatric glaucoma.^22^

Complications in our study include three cases each of hyphaema and cosmetically large blebs; one case each of AC tube block with vitreous, dellen with exposed scleral patch, retracted tube, hypotony and wound dehiscence leading to blebitis and implant extrusion. Other than the case of blebitis and implant extrusion that led to explantation of the valve, the others were considered minor or correctable. A review by Al Torbak *et al*.^23^ concluded that conjunctival dehiscence over the GDD might represent a major risk factor for endophthalmitis which appeared to be a more common problem with children with this type of surgery. The study highly recommended prompt surgical revision of an exposed GDD tube

The Tube vs. Trabeculectomy [TVT]^24^ study demonstrated that the intraoperative complications encountered within the two procedures are not statistically different. However there were more postoperative complications in the trabeculectomy [60%] than in the tube [34%] group [*P* is equal to 0.001] It is not uncommon in Africa to perform a glaucoma surgery with little chance of ever seeing the patient again. In this situation a tube may therefore be preferred over trabeculectomy. In Wilson 2000 and Wilson 2003, there was no statistical difference in complications between trabeculectomy with Mitomycin-C and AGV implant both in short and long term follow-up. It was not possible for us to perform regressive studies because of the few numbers involved and short follow-up time.

The authors' experience with the AGV implant vs. trabeculectomy with MMC suggests a gentle learning curve and easily-acquired skills for surgeons who are already doing filtration surgery. Compared with other alternative glaucoma surgery procedures such as non-penetrating surgery, there are no special instruments or equipment required except the implant itself and the patch graft. A regular instrument tray and set-up for trabeculectomy is adequate to insert the device. The procedure can be performed with relative ease and confidence in most parts of Africa from a technical standpoint. The most significant drawback of the AGV implant is the cost of the implant itself. The cost can be kept a bit lower if a sclera patch can be routinely used to cover the tube. This is easily available from eye banks and can be stored in ethanol at room temperature for up to one year from the date of preservation. In adults with good sclera the tube can be covered by homologous scleral flap.

There are several shortcomings in our study. The sample size is small and results are based on our initial experience. Moreover, the patient follow-up in Africa is relatively inconsistent and hence the number and duration of cases available for study is low. We did not compare our results to other glaucoma procedures; however, this study encourages us to design a randomized controlled trial of AGV vs. trabeculectomy with MMC in this population.

Ahmed valve implantation appears to be safe and effective in refractive pediatric and adult glaucoma in East African patients. A randomized controlled trial comparing the AGV implant with trabeculectomy will determine whether AGV implant surgery could be recommended as a primary surgical option for POAG in East Africa.

## References

[1] Racette L, Wilson MR, Zagwill LM, Weinreb RN, Sample PA (2003). Primary Open Angle Glaucoma in Blacks: A review. Surv Ophthalmol.

[2] Wadhwa SD, Higginbotham EJ (2005). Ethnic Differences in Glaucoma: Prevalence, Management and outcome. Curr Opin Ophthal.

[3] Werschnik C, Schäferhoff C, Wilhelm FW, Wermund TK (2005). The problem of glaucoma in Africa - progress report from Cameroon. Klin Monatsbl Augenheilkd.

[4] Girkin CA (2004). POAG in African Americans. Int Ophthalmol Clin.

[5] Kosoko-Lasaki O, Olivier MM (2003). African American Health Disparity: A glaucoma case study. Int Ophthalmol Clin.

[6] Tielsch JM, Sommer A, Katz J, Royall RM, Quigley HA, Javitt J (1991). Racial variation in the prevalence of POAG: The Baltimore eye Survey. JAMA.

[7] Sommer A, Tielsch JM, Katz J, Quigley HA, Gottsch JD, Javitt JC (1991). Racial differences in the cause-specific prevalence of blindness in east Baltimore. N Engl J Med.

[8] Buhrmann RR, Quigley HA, Barron Y, West SK, Oliva MS, Mmbaga BBO (2000). Prevalence of Glaucoma in a rural East African population. Invest ophthalmol vis sci.

[9] Devgan U, Yu F, Kim E, Coleman AL (2000). Surgical under-treatment of glaucoma in black beneficiaries of medicare. Arch Ophthalmol.

[10] Morris DA, Paracha MO, Shin DH, Kim C, Cha SC, Kim YY (1999). Risk factors for early filtration failure requiring suture release after primary glaucoma triple procedure with adjunctive mitomycin. Arch Ophthalmol.

[11] Lima FE, Magacho L, Carvalho DM, Susanna R, Avila MP (2004). A prospective, comparative study between endoscopic cyclophotocoagulation and the Ahmed drainage implant in refractory glaucoma. J Glaucoma.

[12] Topouzis F, Coleman AL, Choplin N, Bethlem MM, Hill R, Penek WC, Wilson MR (1999). Follow-up of the Original Cohort With the Ahmed Glaucoma Valve Implant. Am J Ophthal.

[13] Shields Textbook of Ophthalmology.

[14] Wilson MR, Mendis U, Paliwal A, Haynatzka V (2003). Long term follow-up of Primary Glaucoma surgery with ahmed glaucoma valve implant verses trabeculectomy. Am J Ophthalmol.

[15] Wilson MR, Mendis U, Smith SD, Paliwal A (2000). Ahmed Glaucoma Valve Implant vs Trabeculectomy in the Surgical Treatment of Glaucoma: A Randomized Clinical Trial. Am J Ophthalmol.

[16] Coleman AL, Hill R, Wilson MR, Tam M (1995). Initial clinical experience with the Ahmed glaucoma valve implant. Am J Ophthalmol.

[17] Ishida K, Netland PA (2006). Ahmed Glaucoma Valve Implantation in African American and White Patients. Arch Ophthalmol.

[18] Huang MC, Netland PA, Coleman AL, Siegner SW, Mosler MR, Hill RA (1999). Intermediate-term Clinical Experience with the Ahmed Glaucoma Valve Implant. Am J Ophthalmol.

[19] Chen TC, Bhatia LS, Walton DS (2005). Ahmed valve surgery for refractory pediatric glaucoma: A report of 52 eyes. J Pediatr Ophthalmol Strabismus.

[20] Morad Y, Donaldson CE, Kim YM, Abdolell M, Levin AV (2003). The ahmed drainage implant in the treatment of pediatric glaucoma. Am J Ophthalmol.

[21] Englert JA, Friedman SF, Cox TA (1999). The ahmed valve in refractory pediatric glaucoma. Am J Ophthalmol.

[22] Djodeyre MR, Peralta Calvo J, Abelairas Gomez J (2001). Clinical evaluation and risk factors of time to failure of Ahmed Glaucoma Valve implant in pediatric patients. Ophthalmology.

[23] Al-Torbak AA, Al-Shahwan S, Al-Jadaan I, Al-Hommadi A, Edward DP (2005). Endophthalmitis associated with the Ahmed glaucoma valve implant. Br J Ophthalmol.

[24] Gedde SJ, Herndon LW, Brandt JD, Budenz DL, Feuer WJ, Schiffman JC (2007). Surgical Complications in the Tube Versus Trabeculectomy Study During the first year of follow-up. Am J Ophthalmol.

